# Management of a Uterosacral Ligament Ectopic Pregnancy With Presence of an Intrauterine Device

**DOI:** 10.1097/og9.0000000000000142

**Published:** 2026-01-22

**Authors:** Sarah Y. Cohen, Barbara Blachut

**Affiliations:** Department of Obstetrics and Gynecology, Washington University School of Medicine, St. Louis, Missouri.

## Abstract

**BACKGROUND:**

Ectopic pregnancy outside of the fallopian tubes is rare and increases the risk of severe maternal morbidity. Extratubal pregnancies frequently pose diagnostic challenges. We describe a case of an ectopic pregnancy implanted on the uterosacral ligament.

**CASE:**

A 30-year-old primigravid patient with an intrauterine device (IUD) presented for management of an ectopic pregnancy without a clearly understood implantation site after an unsuccessful laparoscopic salpingo-oophorectomy at another facility. Repeat laparoscopy identified the pregnancy implanted on the left uterosacral ligament. Pathology confirmed pregnancy tissue, and the patient's β-hCG decreased appropriately.

**CONCLUSION:**

In the presence of an IUD, serial monitoring is imperative given the high risk of ectopic pregnancy. Ectopic pregnancy management requires careful follow-up of pathology and serial β-hCG. Meticulous intraoperative evaluation can help avoid reoperation.

## Abstract 2

Extratubal pregnancies are difficult to diagnose and require careful management and follow-up.

**Teaching Points**

1. Extratubal ectopic pregnancies are difficult to diagnose from blood work and imaging and necessitate very thorough intraoperative evaluation.
2. If a pregnancy occurs with an intrauterine device (IUD) in place, serial monitoring with ultrasound is essential to ensure safe management.

Ectopic pregnancies account for approximately 2% of all pregnancies. More than 95% of cases occur in the fallopian tubes, with only 1% occurring in the abdomen.^1,2^ Ectopic implantation on the uterosacral ligament is an exceedingly rare form of abdominal ectopic pregnancy, with only 11 cases described in the literature over the past 25 years.^3,4^ Ectopic implantation on the uterosacral ligament and other nontubal ectopic pregnancies can pose a diagnostic challenge because of the difficulty of accurate identification on ultrasound. Ectopic pregnancy accounts for 2.7% of pregnancy-related deaths and is the most common cause of death in the first trimester. Delayed diagnosis in nontubal ectopic pregnancies often leads to greater morbidity.^5^ The importance of understanding uterosacral ectopic pregnancies lies in the potential for life-threatening outcomes if not recognized promptly. The present case study describes the approach to the diagnosis and management of an ectopic implantation on the uterosacral ligament in the setting of a copper IUD, highlights the need for accurate diagnosis, and offers guidance on approach for management.

## CASE

The patient provided informed consent for the dissemination of the information included in this article. An exemption was granted by the Washington University IRB.

**Fig. 1.**
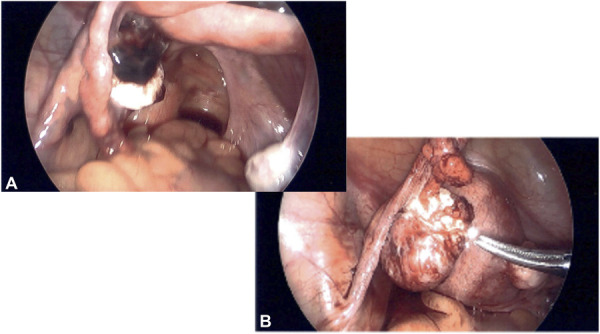
Initial diagnostic laparoscopy and left salpingo-oophorectomy. Left fallopian tube **(A)** and left ovary **(B)**.

This is a case of a 30-year-old woman, gravida 1 para 0, who presented as a transfer from an outside hospital for management of an ectopic pregnancy. The patient had no significant medical history. She had a copper IUD in place for about 2 years. Because of the high risk of ectopic pregnancy in the setting of pregnancy with an IUD in place, the patient had initially been closely followed up at an outside hospital with serial serum β-hCG and ultrasound. The patient's initial β-hCG was 77 international units/L (Table 1). Her serial β-hCG levels rose appropriately for a normal intrauterine pregnancy (more than 50% rise per 48 hours). The patient's initial ultrasound did not demonstrate an intrauterine gestation or adnexal masses. One week later, the patient underwent a subsequent ultrasound that was consistent with an ectopic pregnancy in the left adnexa measuring 6 1/7 weeks with cardiac activity. She elected surgical management after counseling. The patient then underwent a laparoscopic left salpingo-oophorectomy. During the procedure, decision was made to remove the left ovary in addition to the fallopian tube because “it appeared that the ectopic was on the ovary distal, taking up greater than 50% of the ovary” (Fig. 1).

**Table 1.** Monitoring of Ectopic Pregnancy

Day	β-hCG/Event
Day 1*	77
Day 3*	147
Day 4*	Ultrasound 1
Day 8*	1,833
Day 15*	Ultrasound 2
Day 15*	Diagnostic laparoscopy 1
Day 23*	22,681
Day 24*	Ultrasound 3
Day 24	22,635
Day 24	Ultrasound 4
Day 24	Diagnostic laparoscopy 2
Postoperative day 1	5,897
Postoperative week 1	593
Postoperative week 2	56
Postoperative week 3	11
Postoperative week 4	Less than 5

* Performed at an outside hospital laboratory facility.

*Cohen. Uterosacral Ligament Ectopic Pregnancy. O&G Open 202*6.

Surgical pathology was notable for a normal fallopian tube and ovary aside from a large corpus luteum cyst, hematoma, and inflammation. No villi, fetal parts, or vesicles were identified. Moreover, approximately 1 week after the procedure and soon after pathology results, the patient's β-hCG level was rechecked and noted to be significantly increased at 22,681 international units/L. She underwent a repeat ultrasound that showed a persistent ectopic pregnancy with fetal pole measuring consistently with 7 0/7 weeks of gestational age with cardiac activity. At this time, the patient was directed to our hospital for repeat ultrasound and further management.

**Fig. 2.**
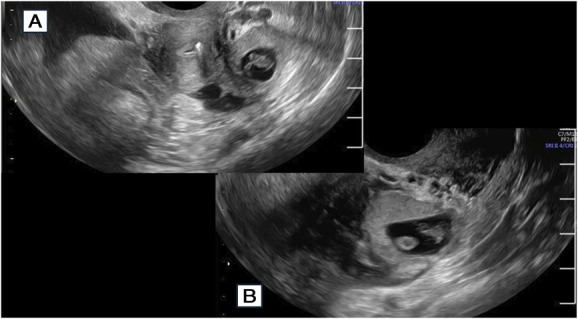
Ultrasonogram images of left adnexal ectopic pregnancy with adjacent uterus. Transverse image **(A)** and longitudinal image **(B)**.

On physical examination, the patient was well appearing. She had no abdominal tenderness. On her pelvic examination, there was no vaginal bleeding, and no masses were palpated. Laboratory values were notable for a hemoglobin of 13.6 g/dL and β-hCG of 22,635 international units/L. Ultrasound revealed concern for a right tubal ectopic pregnancy. The ultrasound noted “right hydrosalpinx that extends to the left side of the uterus posteriorly. Near the end of the hydrosalpinx, there is an ectopic pregnancy with a gestational sac containing a yolk sac and fetal pole with cardiac activity” (Fig. 2 and Videos 1 2, 3. The crown–rump length was consistent with 7 0/7 weeks of gestational age. No free fluid was appreciated.

*Cohen. Uterosacral Ligament Ectopic Pregnancy. O&G Open 202*6.

**Fig. 3.**
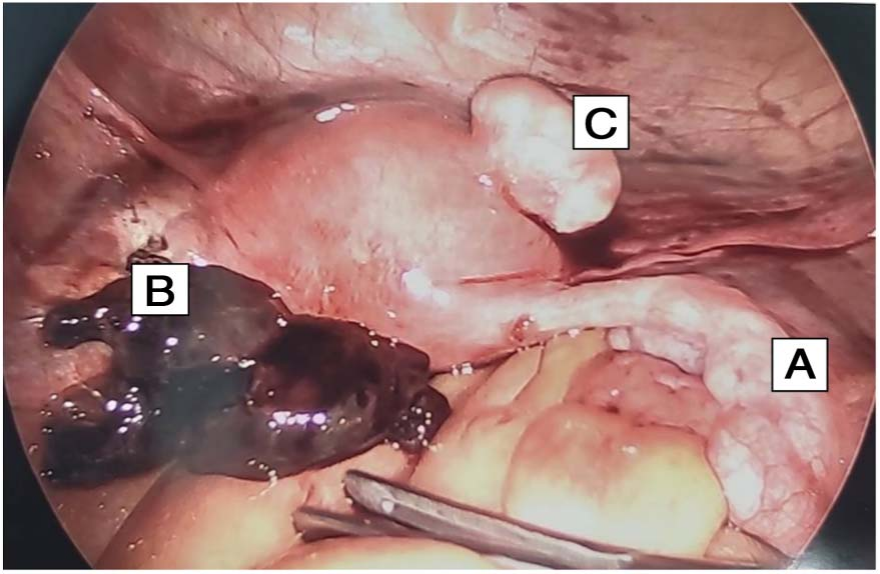
**A.** Right fallopian tube with mild hydrosalpinx. **B.** Hematoma adjacent to site of previous salpingo-oophorectomy. **C.** Small anterior pedunculated leiomyoma.

**Fig. 4.**
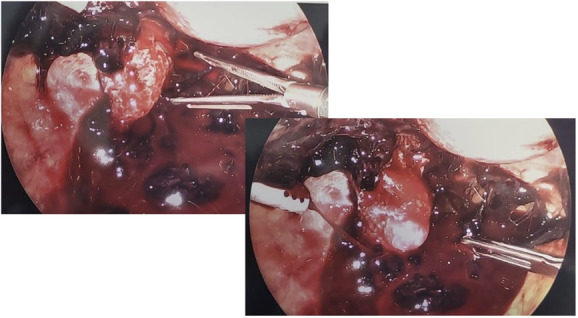
Images of gestational sac adherent to left uterosacral ligament.

**Fig. 5.**
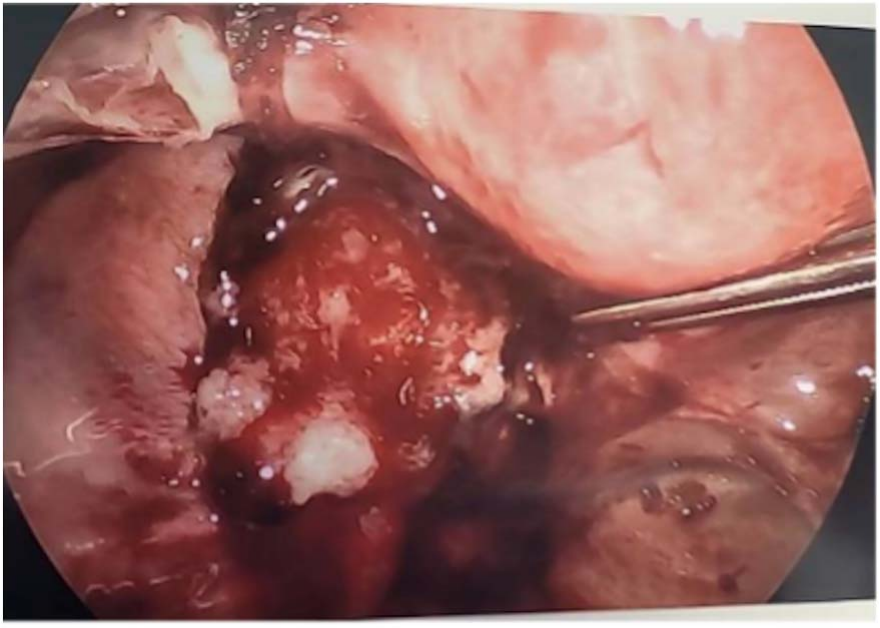
Villous tissue overlying left uterosacral ligament after gestational sac removal.

**Fig. 6.**
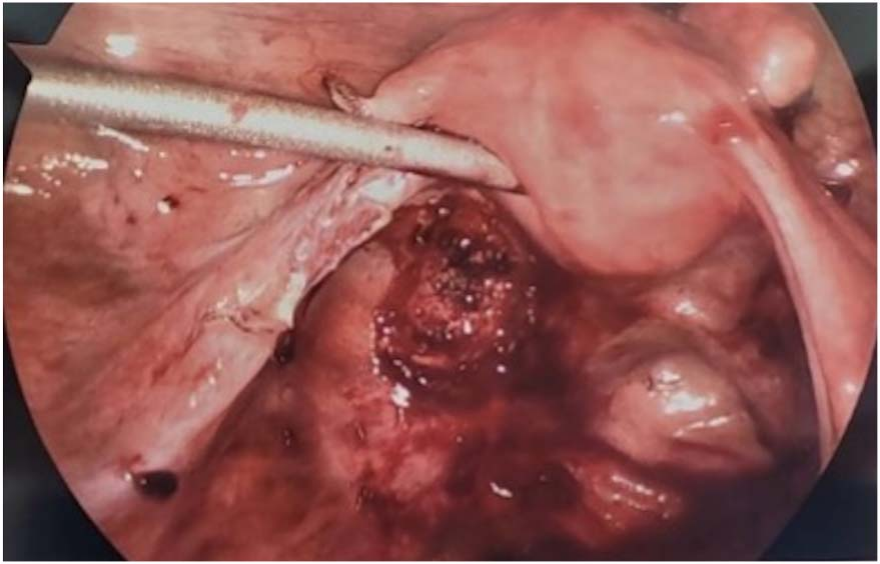
Left uterosacral ligament after removal of ectopic pregnancy and villous tissue.

The patient underwent a diagnostic laparoscopy for evaluation and management of ectopic pregnancy. The uterus appeared normal with a small, approximately 2-cm anterior myoma. The right fallopian tube showed a mild hydrosalpinx, and the right ovary was noted to be normal (Fig. 3). The left tube and ovary were noted to be absent with a moderate-sized clot appreciated at the left adnexa (Fig. 3). Moderate hemoperitoneum was visualized in posterior cul-de-sac (Fig. 4). On closer examination, a bleeding gestational sac–like structure adherent to the left uterosacral ligament was noted (Fig. 4). This structure was bluntly separated from the peritoneum and sent to pathology. Villous material was noted overlying the uterosacral ligament after removal of the sac-like structure (Fig. 5). Copious irrigation and suction were performed. Because of ongoing bleeding from the implantation site, electrocautery and Floseal hemostatic matrix were used to achieve excellent hemostasis (Fig. 6). No other identifiable pregnancy was noted. The patient tolerated the procedure well.

**Video 1.** Longitudinal view of uterus with intrauterine device in place and left-sided ectopic pregnancy in the adnexa. Video created by Hailey Bertani, RDMS, RVT, RDCS, and Sarah Cohen, MD, MPH. Used with permission.

**Video 2.** Transverse view of uterus with ectopic pregnancy in left adnexa. Video created by Hailey Bertani, RDMS, RVT, RDCS, and Sarah Cohen, MD, MPH. Used with permission.

**Video 3.** Transverse view of uterus and ectopic pregnancy, highlighting the close proximity of the right (RT) fallopian tube and the ectopic pregnancy. Video created by Hailey Bertani, RDMS, RVT, RDCS, and Sarah Cohen, MD, MPH. Used with permission.

*Cohen. Uterosacral Ligament Ectopic Pregnancy. O&G Open 202*6.

*Cohen. Uterosacral Ligament Ectopic Pregnancy. O&G Open 202*6.

*Cohen. Uterosacral Ligament Ectopic Pregnancy. O&G Open 202*6.

*Cohen. Uterosacral Ligament Ectopic Pregnancy. O&G Open 202*6.

On postoperative day 1, the patient had a repeat β-hCG level of 5897 international units/L. Pathology results later confirmed the presence of chorionic villi and fetal tissue. The patient was followed up weekly until her β-hCG level was below 5, which occurred approximately 1 month later. She had her copper IUD exchanged in the outpatient setting.

## DISCUSSION

Abdominal ectopic pregnancies are rare, accounting for 1 in 10,000–30,000 pregnancies or about 1 in 100 ectopic pregnancies.^6^ Because of their infrequent occurrence, there has been limited characterization of the associated risks; however, available data suggest that they carry a higher risk of maternal mortality and hemorrhage compared with tubal ectopic pregnancies.^5^ Ectopic implantation on the uterosacral ligament represents an exceptionally rare subtype of abdominal pregnancies in which implantation occurs directly on the uterosacral ligament. Only 11 cases have been described in the literature over the past 25 years.^3,4^

This case illustrates the diagnostic challenges posed by ectopic implantation on the uterosacral ligament—across laboratory evaluation, imaging, and even intraoperative assessment. In this case, β-hCG levels rose at a rate expected for an early intrauterine pregnancy, a finding reported in approximately 20% of ectopic pregnancies.^7^ The abnormal plateau or slow β-hCG rise commonly seen in ectopic pregnancies is thought to result from the inability of the fallopian tube vascular supply to support trophoblast growth. Abdominal implantation sites such as the uterosacral ligament may result in less restriction and support a more “normal” β-hCG trend.^8^ However, published data on β-hCG trends in abdominal ectopic pregnancies remain scarce.

Ultrasound diagnosis of ectopic implantation on the uterosacral ligament may be similarly challenging. In this case, initial sonography suggested a left adnexal ectopic pregnancy. Subsequent imaging after removal of the left fallopian tube and ovary suggested a right tubal ectopic pregnancy. The close proximity of the uterosacral ligament to the adnexa makes this location particularly difficult to identify accurately on imaging.

Intraoperative evaluation may also have limitations, particularly in early pregnancy. During the initial operation, the ovary was presumed to be the site of the pregnancy, likely because of the presence of a large corpus luteum cyst (as noted on pathology) and adjacent blood clot. At the second operation, the true site, a uterosacral ectopic pregnancy, was identified. Although not as rare as ectopic implantations on the uterosacral ligament, ovarian ectopic pregnancies remain very uncommon. This emphasizes the importance of thorough pelvic inspection during surgery, particularly when an extratubal pregnancy is suspected, including careful evaluation of the posterior cul-de-sac, uterosacral ligaments, and peritoneal surfaces. It is imperative to approach a possible ovarian ectopic pregnancy with caution because unilateral oophorectomy is not without long-term risks such as an increased risk of early menopause and a shortened reproductive life span.^9^ Oftentimes, in the absence of active hemorrhage, adjacent ovarian tissue can be preserved with careful removal of the ectopic pregnancy, and oophorectomy can be avoided.^10,11^

Techniques for intraoperative confirmation of successful ectopic pregnancy removal are limited. If the ectopic pregnancy was clearly visualized on ultrasound before surgery, an intraoperative ultrasound can be considered to confirm removal. The use of frozen-section analysis, routine in oncologic surgery, has also been described to analyze endometrial curettings before proceeding with laparoscopy for suspected ectopic pregnancy.^12^ However, there is a dearth of reports regarding its use to confirm the removal of an ectopic pregnancy during surgery. Furthermore, even a full histologic examination is unable to confirm ectopic pregnancy tissue in about 5% of cases.^13^ Although uncommon, frozen section could be carefully considered and discussed with pathology, especially if ectopic removal is suspected but uncertain.

This case underscores the importance of close clinical and imaging follow-up when pregnancy occurs in the setting of an IUD, particularly with ultrasound. It also highlights the need for careful review of pathology findings and postoperative β-hCG monitoring to ensure adequate surgical treatment of ectopic pregnancy and opportunities for improved intraoperative management. This patient's close follow-up enabled timely recognition of the initial ectopic pregnancy despite a normally rising β-hCG, detection of the persistent ectopic pregnancy, and ultimately definitive surgical management. Early identification of atypical ectopic sites is challenging but instrumental to guiding appropriate management and minimizing morbidity.

## Supplementary Material
