# Supplementary figures and images for: Management of a Uterosacral Ligament Ectopic Pregnancy With Presence of an Intrauterine Device
Source: O G Open. 2026 Jan 22;3(1):e142. doi: 10.1097/og9.0000000000000142 (PMC12829682; doi:10.1097/og9.0000000000000142)

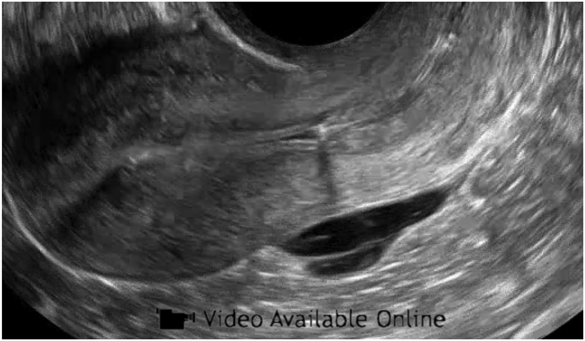

Supplement: Supplementary file 1 [file og9-3-e142-i001.tif]

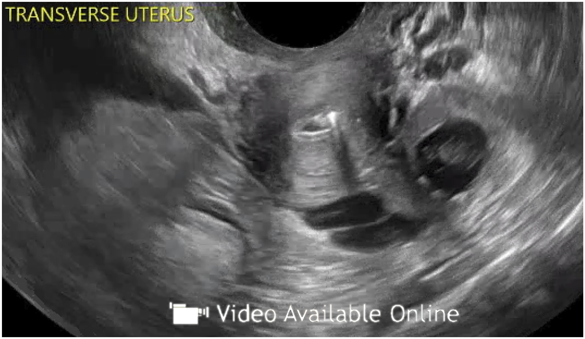

Supplement: Supplementary file 2 [file og9-3-e142-i002.tif]

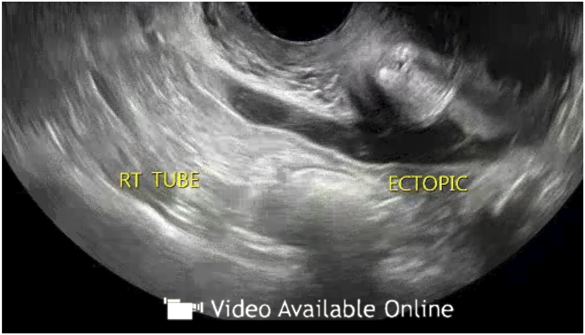

Supplement: Supplementary file 3 [file og9-3-e142-i003.tif]
