# Supplementary material for: Management of a Uterosacral Ligament Ectopic Pregnancy With Presence of an Intrauterine Device
Source: O G Open. 2026 Jan 22;3(1):e142. doi: 10.1097/og9.0000000000000142 (PMC12829682; doi:10.1097/og9.0000000000000142)
Supplement: Supplementary file 4 [file og9-3-e142-s001.pdf]

NOTICE: This document contains correspondence generated during peer review and subsequent revisions but before transmittal to production for composition and copyediting:

- Comments from the reviewers and editors (email to author requesting revisions)
- Response from the author (cover letter submitted with revised manuscript)*

**The corresponding author has opted to make this information publicly available.*

Personal or nonessential information may be redacted at the editor's discretion.

Questions about these materials may be directed to the *O&G Open* editorial office: obgyn@greenjournal.org.

View External Correspondence:

Close

Date: Sep 22, 2025
Description: Transfer Letter
To:
From: "The Green Journal" em@greenjournal.org - Obstetrics & Gynecology
Subject: Transfer Letter
Attachment(s):

Ref.:Case of A Uterosacral Ligament Ectopic Pregnancy with Presence of an IUD
Sarah Yael Cohen

I am transferring the above article to you because I believe it is well-suited to your publication.

Please see the Decision letter included below for our editors' and reviewers' comments on the article.

Regards,

Obstetrics & Gynecology

Obstetrics & Gynecology Decision Letter:

To:
From: "The Green Journal" em@greenjournal.org
Subject: Your Submission ONG-25-1635
09/12/2025

RE: ONG-25-1635 ("Case of A Uterosacral Ligament Ectopic Pregnancy with Presence of an IUD")

Dear Dr. Cohen:

Your manuscript has been reviewed by the Editorial Board and expert reviewers. Based on the Reviewers' comments and the Editors' reading of your manuscript, we are not able to accept it for publication in Obstetrics & Gynecology. However, the Editors have identified your work as important and would like to invite you to revise your manuscript for consideration for publication in O&G Open (open.greenjournal.org), a new open-access journal within the American College of Obstetricians and Gynecologists' portfolio of journals.

Transfer offers are only extended to a small subset of authors, and the majority of submissions are not sent for additional peer review. O&G Open offers low article processing charges and a discount for ACOG members. More information is available at https://journals.lww.com/ogopen/pages/default.aspx.

Prior to submitting your response to this transfer offer, please note the following:

* Articles accepted by O&G Open will be available online at open.greenjournal.org, and promoted and disseminated by Obstetrics & Gynecology.

* Agreeing to transfer your submission to O&G Open is not a guarantee of acceptance and publication. The manuscript will be evaluated according to the normal standards of the Editors and must be revised appropriately.

* By transferring your submission to O&G Open, you are agreeing to be responsible for paying the article processing charge, as determined by the publisher, if the evaluation of your final submission results in acceptance. Please note that prices vary by article type, and are posted at https://journals.lww.com/ogopen/Pages/Open-Access.aspx. First and corresponding authors who are current ACOG members are eligible for a 25% discount.

Instructions for transferring:

* After initiating the transfer by clicking the Accept link below, authors will have their submissions returned to them through O&G Open and will have 21 days to upload their point-by-point cover letter to the Reviewers' comments, a revised manuscript that displays the tracked changes, and any related files.

* When you submit your manuscript in O&G Open's Editorial Manager, please enter your Article Title as "TRANSFER: [Title]" to help the Editor identify the submission.

* Please read the Revision Checklist at https://journals.lww.com/greenjournal/Documents/RevisionChecklist_Authors.pdf and make the applicable edits to your revised manuscript.

* Your username and password for the O&G Open Editorial Manager site (URL: https://www2.cloud.editorialmanager.com/ogopen/) are the same credentials used to access the Obstetrics & Gynecology Editorial Manager site: Your username is: ******** / ********.

To accept this invitation to transfer your manuscript and accompanying peer reviews to O&G Open, please click the "Agree to Transfer" link:

* Accept Transfer - ********

You may also decline to transfer by clicking here:

* Decline Transfer - ********

Please note that this offer to transfer will expire in 14 days.

Sincerely,

The Editors of Obstetrics & Gynecology

- - - - - - - - - - - - - - - - - - - -

EDITORIAL OFFICE COMMENTS:

Figures 1-4: Please upload a higher resolution version of these figures. Please upload as individual figure files on Editorial Manager. The figure legends can remain in the manuscript.

REVIEWER COMMENTS:

Reviewer #1:

This is an interesting and valuable case report describing a patient with a uterosacral ligament (USL) ectopic pregnancy, which was initially not recognized and underwent suboptimal early management.

The report effectively highlights important clinical pitfalls in both the diagnosis and surgical management of this rare condition. It underscores the diagnostic challenges associated with non-tubal ectopic pregnancies. This report contributes to the limited literature on USL pregnancies by illustrating an unusual clinical course—including significantly elevated and rising hCG levels consistent with an intrauterine pregnancy, and progression to a relatively advanced gestational duration. Additionally, the report emphasizes surgical missteps and gaps in follow-up that offer valuable learning points for clinicians.

While there are a few minor semantic issues and some unanswered questions, the manuscript addresses a rare yet clinically relevant entity and deserves publication.

I have a few specific suggestions and questions:

Page 1: Please consider rephrasing the phrase "management of an ectopic pregnancy of unknown location." The term "unknown location" may create confusion given the formal use of "pregnancy of unknown location" (PUL) in clinical terminology.

Page 2: Consider rewording "complicated by the presence of" to "in the setting of" for smoother readability.

Page 2: Instead of stating that the "initial ultrasound was normal," please clarify that the ultrasound did not identify a definitive intrauterine or ectopic pregnancy (e.g., "the initial ultrasound did not demonstrate an intrauterine gestation or adnexal masses").

It would significantly strengthen the report if intraoperative images from the initial surgery—especially showing the left ovary—could be included. Additionally, it would be helpful to include a brief discussion on ovarian preservation and the management considerations specific to ovarian ectopic pregnancies.

It would enhance the educational value to provide sonographic images from your institution, ideally demonstrating the relationship of the ectopic pregnancy to the uterus and adnexa. If available, cine clips could be particularly useful for the online version of the Journal.

Could you elaborate on the clinical course and management between days 8 and 23? This interval seems clinically relevant and might help readers better understand the decision-making process and evolution of the case. In case the patient was lost to follow-up during that critical time frame - please mention steps undertaken to contact the patient.

Reviewer #2:

Excellent presentation of a very difficult case. I would like to offer a couple of suggestions.

First, are there ways intraoperatively to confirm removal of ectopic pregnancy (such as frozen section, etc)? This maybe worth putting in the discussion section.

Second, it should be pointed out that the first surgery may have missed the ectopic due to its smaller size. It is not clear the timeline between the first and second surgery and clearly stating that would be helpful.

Otherwise, this is an excellent presentation.

Close

Submission of manuscript, “Management of A Uterosacral Ligament Ectopic Pregnancy with Presence of an IUD”

The Editors
O&G Open

Dear Editors:

On behalf of myself and my co-author, I am pleased to submit our manuscript, “Management of A Uterosacral Ligament Ectopic Pregnancy with Presence of an IUD,” for consideration for publication as a case report in *O&G Open*. Each author participated actively in drafting the manuscript, editing, and approving the final, submitted version. None of the authors has a financial or other conflict of interest. The manuscript is not under consideration elsewhere and will not be submitted elsewhere unless a final negative decision is made by the Editors of *O&G Open*. This submission is a transfer from *Obstetrics & Gynecology* and thus, below you will see a point-by-point response to the peer reviewers’ comments.

- “Figures 1-4: Please upload a higher resolution version of these figures. Please upload as individual figure files on Editorial Manager. The figure legends can remain in the manuscript.”
    - Response: Higher resolution images were uploaded.
- “Page 1: Please consider rephrasing the phrase "management of an ectopic pregnancy of unknown location." The term "unknown location" may create confusion given the formal use of "pregnancy of unknown location" (PUL) in clinical terminology.”
    - Response: Text edited to reflect this suggestion.
- “Page 2: Consider rewording "complicated by the presence of" to "in the setting of" for smoother readability.”
    - Response: Text edited to reflect this suggestion.
- “Page 2: Instead of stating that the "initial ultrasound was normal," please clarify that the ultrasound did not identify a definitive intrauterine or ectopic pregnancy (e.g., "the initial ultrasound did not demonstrate an intrauterine gestation or adnexal masses").”
    - Response: Text edited to reflect this suggestion.
- “It would significantly strengthen the report if intraoperative images from the initial surgery—especially showing the left ovary—could be included. Additionally, it would be helpful to include a brief discussion on ovarian preservation and the management considerations specific to ovarian ectopic pregnancies.”
    - Response: Images were requested and obtained from outside hospital facility. An additional figure was added to illustrate the intra-operative findings from the initial surgery.
    - Response: Information was added to highlight the risks of oophorectomy and that the limited data available on ovarian ectopic pregnancies suggests management does not require oophorectomy.
- “It would enhance the educational value to provide sonographic images from your institution, ideally demonstrating the relationship of the ectopic pregnancy to the uterus and adnexa. If available, cine clips could be particularly useful for the online version of the Journal.”
    - Sonographic images and video clips were added
- “Could you elaborate on the clinical course and management between days 8 and 23? This interval seems clinically relevant and might help readers better understand the decision-making

process and evolution of the case. In case the patient was lost to follow-up during that critical time frame - please mention steps undertaken to contact the patient."

  - Response: The Table was updated to reflect all hCG values, ultrasounds and the two procedures to improve the ease with which the reader could follow the timeline of the management. It was also clarified that hCG was rechecked after pathology resulted and indicated an ectopic pregnancy was not removed.

- "First, are there ways intraoperatively to confirm removal of ectopic pregnancy (such as frozen section, etc)? This maybe worth putting in the discussion section."
  - Response: Added a few sentences on the limited use of frozen section as well as noted that intra-operative ultrasound can be used to confirm removal in the case where the ectopic was clearly seen pre-operatively.
- "Second, it should be pointed out that the first surgery may have missed the ectopic due to its smaller size. It is not clear the timeline between the first and second surgery and clearly stating that would be helpful."
  - Response: The Table was updated to more clearly illustrate the timeline events including the interval between surgeries.

This study was deemed non-human subjects research and exempt from IRB by the Washington University School of Medicine Institutional Review Board. The patient signed a HIPAA waiver for release of health information. The patient signed an authorization for release of media information, which covered release of protected health information for the purposes of this case study.

We look forward to your comments and critique of the manuscript.

If you have any questions about the manuscript, please do not hesitate to contact us.
Thank you for your consideration.

Sincerely,
Sarah Cohen, MD, MPH
